# Robust desynchronization of Parkinson’s disease pathological oscillations by frequency modulation of delayed feedback deep brain stimulation

**DOI:** 10.1371/journal.pone.0207761

**Published:** 2018-11-20

**Authors:** Mohammad Daneshzand, Miad Faezipour, Buket D. Barkana

**Affiliations:** 1 D-BEST Lab, Departments of Computer Science and Engineering and Biomedical Engineering, University of Bridgeport, Bridgeport, CT, United States of America; 2 Department of Electrical Engineering, University of Bridgeport, Bridgeport, CT, United States of America; University Paris 6, FRANCE

## Abstract

The hyperkinetic symptoms of Parkinson’s Disease (PD) are associated with the ensembles of interacting oscillators that cause excess or abnormal synchronous behavior within the Basal Ganglia (BG) circuitry. Delayed feedback stimulation is a closed loop technique shown to suppress this synchronous oscillatory activity. Deep Brain Stimulation (DBS) via delayed feedback is known to destabilize the complex intermittent synchronous states. Computational models of the BG network are often introduced to investigate the effect of delayed feedback high frequency stimulation on partially synchronized dynamics. In this study, we develop a reduced order model of four interacting nuclei of the BG as well as considering the Thalamo-Cortical local effects on the oscillatory dynamics. This model is able to capture the emergence of 34 Hz beta band oscillations seen in the Local Field Potential (LFP) recordings of the PD state. Train of high frequency pulses in a delayed feedback stimulation has shown deficiencies such as strengthening the synchronization in case of highly fluctuating neuronal activities, increasing the energy consumed as well as the incapability of activating all neurons in a large-scale network. To overcome these drawbacks, we propose a new feedback control variable based on the filtered and linearly delayed LFP recordings. The proposed control variable is then used to modulate the frequency of the stimulation signal rather than its amplitude. In strongly coupled networks, oscillations reoccur as soon as the amplitude of the stimulus signal declines. Therefore, we show that maintaining a fixed amplitude and modulating the frequency might ameliorate the desynchronization process, increase the battery lifespan and activate substantial regions of the administered DBS electrode. The charge balanced stimulus pulse itself is embedded with a delay period between its charges to grant robust desynchronization with lower amplitudes needed. The efficiency of the proposed Frequency Adjustment Stimulation (FAS) protocol in a delayed feedback method might contribute to further investigation of DBS modulations aspired to address a wide range of abnormal oscillatory behavior observed in neurological disorders.

## Introduction

Parkinson’s disease (PD) is a neurodegenerative disorder associated with altered firing activity of the Basal Ganglia (BG) nuclei causing symptoms such as rigidity, tremor and akinesia. The intervention of the nervous system through electrical pulses of Deep Brain Stimulation (DBS) regulates the neuronal activities in PD [1, 2]. The effectiveness of DBS is argued to be related to the elimination of the rhythmic activity seen in PD by reducing the synchronization in the beta band (13–35 Hz) and by increasing it in the gamma band (35–70 Hz) [3–5]. Subthalamic Nucleus (STN) or Globus Pallidus interna (GPi) nuclei are the common targets for DBS [6], in which both targets have shown to yield great outcomes in the treatment of dyskinesia, motor fluctuation and rigidity [7]. To achieve the optimum outcome of DBS, we must consider the symptoms of the patient, the neural pathways targeted, and the stimulation parameters [8–11]. Clinical DBS waveforms are consisted of a rectangular high amplitude cathodic phase followed by a low amplitude anodic phase, however, other studies have suggested sinusoid and Gaussian pulses where Gaussian DBS are shown to reduce the energy usage of the device by 50% [8, 10]. Reducing the consumed energy of the DBS signal can increase the battery life and eliminate the costly replacement surgeries [12, 13]. In addition, introducing a delay between the cathodic and anodic phases of the DBS pulse contributes to better desynchronization and energy efficiency and harvesting of the process [8, 14–18].

Neuronal activities of mammalian forebrain tend to show oscillatory behaviors in a certain range of frequencies [19]. Moreover, different symptoms of PD are associated with various frequency ranges such as bradykinesia which is related to beta oscillation while gamma band oscillations are often associated with prokinetic symptoms [3]. Axial symptoms of PD such as gait, postural stability [20] and speech are better treated with Low Frequency Stimulation (LFS) in the range of (60–80 Hz), while High Frequency Stimulation (HFS) is suitable for tremor, rigidity and bradykinesia [21–23]. Oscillatory properties of the neuronal activity are mostly ameliorated by HFS [24, 25], which based on some theories, is due to the locking of the neuronal firing discharge time to the frequency of stimulation [26]. Many studies have shown that the inhibition induced by HFS alters the mean firing rate of the STN neurons and alters the neurotransmitter release and antidromic activation of the BG cells [25, 27, 28]. Considering the high energy cost of HFS and various therapeutic results of the stimulation frequency, new DBS parameterization could combine HFS and LFS. The mixed mode of DBS frequencies can exceedingly target various symptoms of PD [29]. For instance, LFS has shown to improve the axial symptoms of PD such as postural instability, gait dysfunction, swallowing and speech problems, while HFS can address motor symptoms, bradykinesia and rigidity [29, 30].

Improving the symptoms while reducing the side effects cannot cope with the shorter temporal dynamics of PD in an open loop stimulation paradigm [31]. Therefore, there is a need for dynamic stimulation systems such as closed loop or delayed feedback DBS, that are capable of continually adopting the stimulus based on the aggregated neuronal firing patterns. It has been shown that closed loop DBS ameliorates akinesia and abnormal Cortico-BG discharges [32], improves therapeutic efficiency, increases battery lifespan, decreases tissue damage, and adjusts the oscillatory patterns [11, 33, 34]. Closed loop models usually use the Local Field Potential (LFP) of the targeted region as the control variable since it is highly correlated with changes in the motor system [23, 35, 36]. LFP is then filtered and analyzed to be fed in a feedback algorithm. The decision of the feedback algorithm will set the next parameters for the DBS signal. For higher performance, the stimulation amplitude is reduced according to the amplitude of the filtered LFP signal [17] [37].

We propose a new frequency adaptation stimulus according to the variation of LFP in a closed loop model. Our protocol adjusts the frequency of stimulation according to the level of synchrony observed by the LFP signal. For instance, HFS is only applied at the peaks of LFP signal where the synchronization is relatively high and the stimulation frequency declines as the synchronization level reduces. Closed loop adjustment of the frequency of stimulation shows better desynchronization while being energy efficient [32]. In addition, frequency adaptation has more therapeutic effects since various symptoms of PD correlate with different ranges of stimulation frequencies [29, 38].

Biologically inspired models capture the characteristics of various nuclei, however, these models are computationally complex. Low dimensional models, on the other hand, reduce the computational costs while the lack of physiological implications make the LFP estimation and feedback control more challenging. To reduce the computational cost while considering neural interconnections and properties of each nuclei within the BG network, we propose a 3 dimensional model based on the Izhikevich formulation [39]. Low cost computation of this model guarantees the simulation of large neuronal population. We also consider the synaptic connections within all neurons based on more realistic models to examine the synchrony in the Cortico-BG network along with LFP assessments. Our model is able to generate the membrane voltages of the BG neurons, temporal firing patterns, and synchrony dynamics seen in experimental recordings [40].

## Methods

### BG model

**Fig 1:**
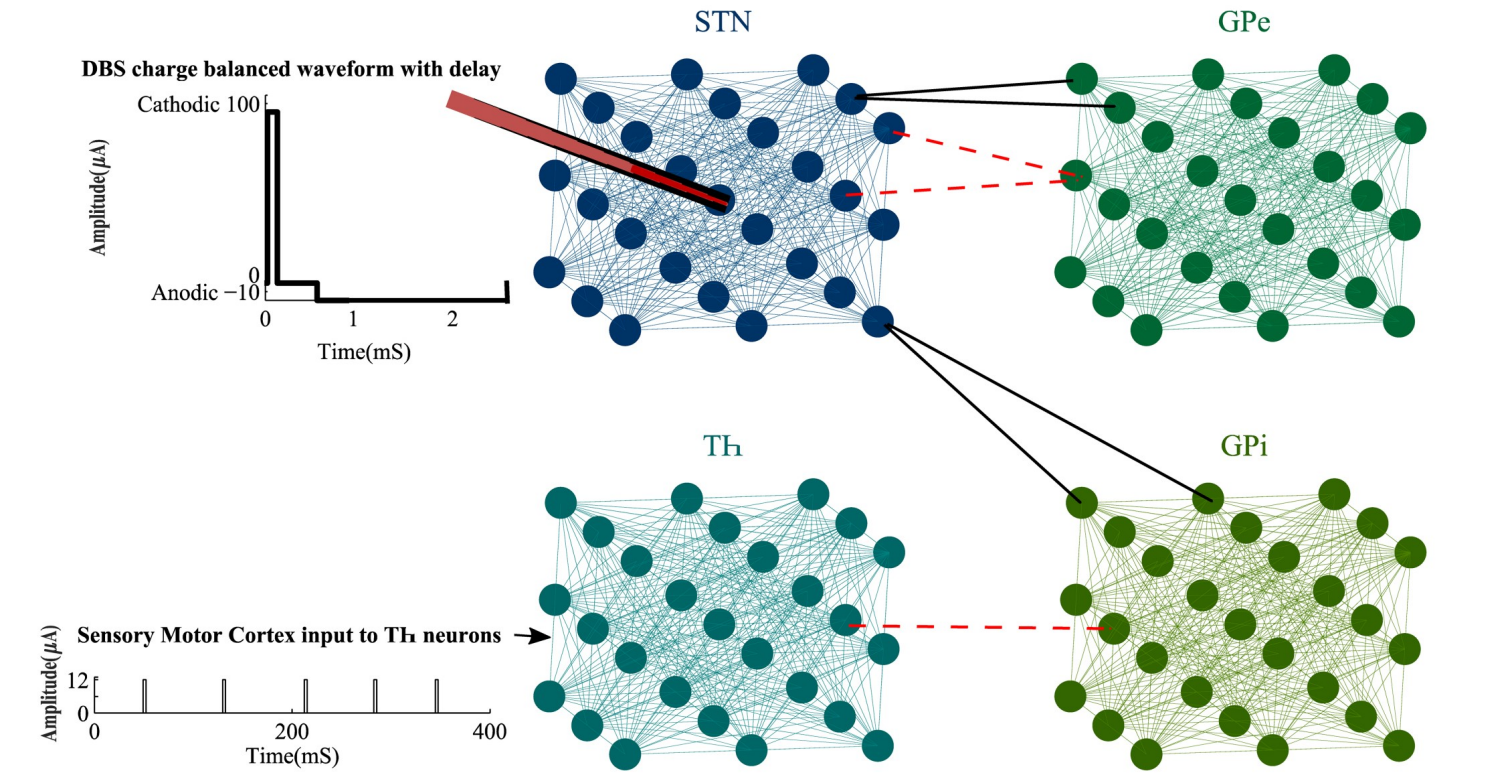
Proposed BG network. The BG network consists of 4 types of nuclei placed in cubic space with internal connections between each type. These nuclei are connected through excitatory (black lines) and inhibitory (red dashed lines) synopsis. The charge balanced DBS signal is applied at the centric neuron of the STN population and is added with an interphase delay to provide better desynchronization results while activating silent neurons. Th neurons received a pulse train representing the sensory motor cortex input to the BG network. For clarity, only 27 neurons in each subpopulation are shown here, however, the network is able to model large populations as well (1000 neurons in each nucleus).

Thalamic (Th), Subthalamic Nucleus (STN), Globus Pallidus externa, and interna (GPe and GPi) are the main neuronal types of our BG model. Each nucleus has a population of 125 neurons with their interconnections. These neuronal subpopulations are aligned in a 5×5 symmetric cubic space, as shown in Fig 1. Each STN neuron has excitatory connections to 2 GPe and 2 GPi neurons. GPe neurons have inhibitory connections to 2 STN neurons and finally, there is one inhibitory synopsis from each GPi to a Th neuron [41]. We considered a local field of connections between all pairs of neurons within each nucleus (Fig 1), to match the local connectivity developed by hippo-campus studies [42]. It has been shown that glutamatergic synapses exist in the STN neurons [43]. The connections of Globus Pallidus neurons are mediated by *GABA*_*A*_ receptors [44, 45] and local interneuron synapses control the Thalamic circuitry [46]. Therefore, we considered the excitatory coupling between neurons in each subpopulation which is missing in many computational models of the BG [11, 17, 33]. These synaptic connections within each nucleus were obtained by the following equation as a function of the membrane and resting state voltages *V*^*j*^ and $E_{S}^{j}$, respectively.

$$I_{S}^{j}=g_{S}(V^{j}-E_{S}^{j})\sum_{i=1}^{N}W_{ij}S_{S}$$ (1)

where $I_{S}^{j}$ is the total synaptic currents from all neurons of a specific nucleus to neuron *j*. The membrane conductance *g*_*S*_ was set to 1.5, 3.5, and 10 for the Th, STN, and GP populations, respectively, to assure the desired connections. In order to reflect the strength of the connections within each nucleus, we account the synaptic weights *W* based on the distance between each pair of neurons. Therefore, $\sum_{i=1}^{N}W_{ij}$ in Eq 1 denotes the sum of all weights from *N* neurons in the population to neuron *j*. These weights were measured as follows.

$$W_{ij}=e^{\frac{{-\|n_{i}-n_{j}\|}^{2}}{{2\sigma }^{2}}}$$ (2)

In Eq 2, ‖*n*_*i*_−*n*_*j*_‖^2^ is the Euclidean distance between neuron *i* and neuron *j*. The parameter *σ* was set to a small value to ensure that relatively far neurons receive weak and negligible connections from each other as opposed to primary projecting neurons and interneurons, where stronger connections are needed. The synaptic dynamic *S*_*S*_ in Eq 1 was defined by a first order process to reduce the computational cost of a large network.

$$\frac{dS_{S}}{dt}=-\alpha_{S}S_{S}+\delta (t-T)$$ (3)

where *α*_*S*_ and *T* represent the reverse potential and the time of presynaptic spikes, respectively [47]. The excitatory and inhibitory synapses between different BG cells were defined by Eq 4. *S*_*j*_ in this Equation stands for the summation of all presynaptic dynamics. In case of inhibitory connections from GPe to STN, *S*_*j*_ consists of 2 presynaptic currents, while *S*_*j*_ would have only one presynaptic current for the GPi-Th connections.

$$I_{syn}^{i\to j}=g_{ij}S_{i}(V^{j}-E_{syn}^{j})$$ (4)

We also considered a random pulse train *I*_*SMC*_, modeling the aggregated inputs from sensory motor cortex to Th (Fig 1). The amplitude of this current was set to 12 *μA* with pulse width of 2.8 *mS*. After general initialization of our BG model, the governing membrane voltage equation for each neuron type was achieved by an extension over the Izhikevich spike formulations [39].

#### Th neurons

The output of the BG network is the projection of the GPi to Th neurons and the firing of Th is shown to be spontaneous, however, the increase in the input currents to Th (inhibitory connections from GPi), alleviates the firing rate. In order to model the large population of 125 Th neurons, we used the reduced order Izhikevich tonic model [39]. The dynamics of a single Th neuron is then formulized by a set of 2 equations.

$$\frac{dV_{Th}}{dt}=0.04{V_{Th}}^{2}+5V_{Th}+140-u_{Th}+I_{S}^{Th}+I_{SMC}-I_{syn}^{GPi\to Th}$$ (5)

$$\frac{du_{Th}}{dt}=a(b{V_{Th}}^{2}-u_{Th})$$ (6)

The membrane voltage of each Th neuron consists of synaptic currents from other Th neurons ($I_{S}^{Th}$), inhibitory synapse from a GPi neuron ($I_{syn}^{GPi\to Th}$) and the sensory motor cortex current (*I*_*SMC*_). The auxiliary variable *u*_*Th*_ is set to a reset value (c) after every peak of *V*_*Th*_. Parameters *a* and *b* reflect the recovery rate and sensitivity of *u*_*Th*_, respectively.

#### STN neurons

Depolarizing currents elicit Action Potentials (APs) in the STN neurons with a rebound burst after the hyperpolarizing current is off [48]. STN also shows synchronized bursting which leads to rhythmic patterns. To capture these characteristics of the STN neurons, the Equation below is defined.

$$\frac{dV_{STN}}{dt}=0.04{V_{STN}}^{2}+5V_{STN}+140-u_{STN}+I_{S}^{STN}+I_{N}^{STN}-I_{syn}^{GPe\to STN}+I_{appSTN}+e^{-D}I_{DBS}$$ (7)

where $I_{S}^{STN}$ is the total synaptic current from other STN neurons denoted by Eq 1 and $I_{syn}^{GPe\to STN}$ is the inhibitory synapse from 2 GPe neurons to one STN. These inhibitory connections are weakened by deprivation of the dopaminergic cells in PD. Other regions of the brain send synaptic inputs to the STN neurons which is defined by $I_{N}^{STN}$ and is used to keep the firing rate of the STN neurons in the experimentally observed frequency range [49]. Moreover, we added a constant current *I*_*app*_ to switch from healthy conditions to PD. Synchronous behavior in STN firing appears with the smaller synaptic currents from the GPe to STN neurons. The first order synaptic dynamic (Eq 3) was used to model the GPe-STN connection which is believed to act as a pacemaker generating oscillations in PD [50]. This allows the model to generate the healthy firings while being able to maintain the GPe-STN connection which could be a source for maintaining the synchronous dynamics.

The DBS signal is consisted of a Cathodic phase with amplitude of 100 *μA* and duration of 0.2 *mS* followed by a 2 *mS* Anodic phase with amplitude of -10 *μA*. This biphasic stimulation results in net charge of zero, injected to the tissue and prohibits the tissue damage [33, 34]. We also added a delay of 0.5 *mS* between the Cathodic and Anodic phases [8, 16] as shown in Fig 1. The longer interphase delay significantly improves the desynchronization process and it has been shown that the delay length is related to the activation of silent neurons and entrainment of bursting neurons [12]. Since the DBS was targeted at the centric neuron in the STN cubic population (Fig 1), its efficiency decreases according to the distance of other neurons to the stimulation electrode. Commonly, the effects of stimulation on neuronal firing patterns decay as a function of distance between the electrode and the desired neuron [6]. The term *e*^−*D*^ in Eq 7 provides an exponentially debilitating effect on how each neuron is influenced by the DBS current, where *D* is the Euclidean distance between the neuron and the electrode. *u*_*STN*_ incorporates an Ordinary Differential Equation ODE such as Eq 6 with different adjusting parameters stated in Table 1.

**Table 1:** Nominal values of the BG model parameters.

	*α*_*S*_	*a*	*b*	*c*	*d*	*I*_*app*_(*μA*)
**Th**	0.5	0.02	0.2	-65	5	0
**STN**	0.5	0.01	0.27	-65	8	1
**GPe**	0.3	0.2	0.26	-65	0	0.2
**GPi**	0.3	0.2	0.26	-65	0	0.3
**Synaptic Currents**	*g*_*GPe*→*STN*_ = 1.5	*g*_*STN*→*GPe*_ = 2.5	*g*_*GPe*→*GPe*_ = 1.5	*g*_*STN*→*GPi*_ = 2.5	*g*_*GPi*→*GPi*_ = 1.5	*g*_*GPi*→*Th*_ = 2.3
	*E*_*GPe*→*STN*_ = -85	*E*_*STN*→*GPe*_ = 0	*E*_*GPe*→*GPe*_ = -65	*E*_*STN*→*GPi*_ = 0	*E*_*GPi*→*GPi*_ = -65	*E*_*GPi*→*Th*_ = -65

#### GPe and GPi neurons

GPe and GPi neurons have similar properties with continuous repetitive firing patterns. There are slight differences in afferent connections of GPe and GPi neurons causing disparities in their synaptic currents and membrane voltages [51]. To address these slight variations of GPe and GPi neurons, we adjusted the parameters of Izhikevich firing patterns along with modification of $I_{N}^{GPe}$ and $I_{N}^{GPi}$. Although this parametrization affects the firing rates, the spiking patterns of GPe and GPi neurons were continuously repetitive and bistable [39]. The equations below define the membrane voltage of the GPe and GPi neurons in our BG network.

$$\frac{dV_{GPe}}{dt}=0.04{V_{GPe}}^{2}+5V_{GPe}+140-u_{GPe}+I_{S}^{GPe}+I_{N}^{GPe}+I_{appGPe}-I_{syn}^{STN\to GPe}$$ (8)

$$\frac{dV_{GPi}}{dt}=0.04{V_{GPi}}^{2}+5V_{GPi}+140-u_{GPi}+I_{S}^{GPi}+I_{N}^{GPi}+I_{appGPi}-I_{syn}^{STN\to GPi}$$ (9)

The inhibitory connections between the GPe neurons ($I_{syn}^{GPe\to GPe}$) are considered in $I_{N}^{GPe}$ according to Eq 1. $I_{N}^{GPi}$ was set higher than $I_{N}^{GPe}$ (see Table 1) to ensure higher firing rates of the GPi neurons shown in experimental recordings [26]. Again, *u*_*GPe*_ and *u*_*GPi*_ were adjusted via Eq 6 to obtain the burst firings seen in PD.

### Feedback loop

**Fig 2:**
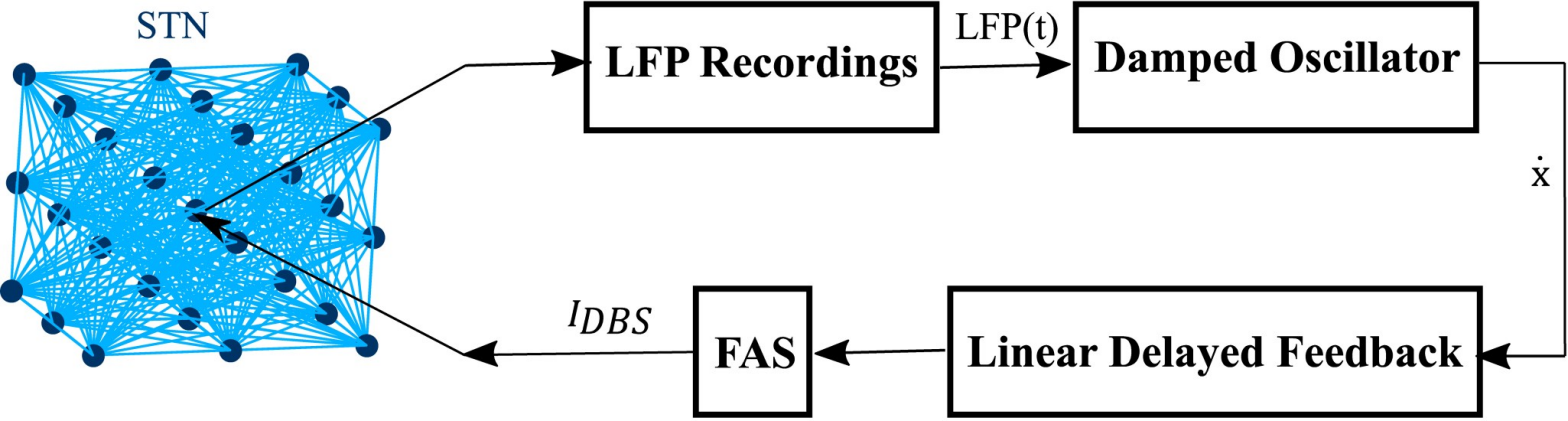
Feedback loop. The LFP is recorded from the center of the STN population and then filtered with a damped oscillator. The result is shifted through a linear delayed feedback block and is used to adjust the DBS current. The frequency of the biphasic DBS signal is adjusted linearly based on the amplitude of $\dot{x}(t)$. The larger the amplitude of $\dot{x}(t)$ is, the higher the frequency of the DBS biphasic pulses will be.

Rhythmic oscillation of the STN neurons interacting with the GPe cells has been observed in PD [52]. This rhythmic nature can be captured by the LFPs of the STN neurons. We used the same location as the DBS electrode was targeted to measure the LFP of the STN neurons, according to the following Equation [17, 53].

$${LFP}_{STN}(t)=\frac{R}{4\pi }\sum_{i=1}^{N}\frac{I_{STNi}(t)}{D_{ic}}$$ (10)

where *R* is the extracellular resistance set to 1, assuming to be homogenous throughout the population. *D*_*ic*_ is the Euclidean distance between neuron *i* and the center of population where the LFP recording electrode is placed (Fig 1). and *I*_*STNi*_(*t*) is composed of all currents on the left-hand side of Eq 7 for the *i*^*th*^ STN neuron. The LFP signal is then filtered using a damped oscillator as follows.

$$\ddot{x}+\omega \dot{x}+\omega^{2}x=K_{S}{LFP}_{STN}(t)$$ (11)

where *ω* denotes the frequency of oscillation and is approximated at 62 $\frac{rad}{sec}$ since the period of each oscillation is around 100 *mS* $\left(\omega =\frac{2\pi }{T}\right)$. *K_S_* is a scaling coefficient set to 0.01 in this filter. The output of the damped oscillator is often delayed due to the filtering process. Thus, the feedback stimulator signal *FS*(*t*) is defined by shifting $\dot{x}$ by half of the period of oscillation. This is essentially a linear delayed feedback used in closed loop stimulations [16].

$$FS(t)=I_{DBS}(K{LFP}_{m}(t)t)$$ (12)

$${LFP}_{m}(t)=\dot{x}\left(t-\frac{T}{2}\right)-\dot{x}(t)$$ (13)

where *LFP*_*m*_(*t*) is the filtered and delayed LFP signal. $T=\frac{\omega }{2\pi }$ is the period of oscillation and *K* is the feedback gain set to 2. The *FS*(*t*) acts as a linear delayed feedback control to adjust the frequency of the stimulation signal *I*_*DBS*_. We introduce a Frequency Adjustment Stimulation (FAS) method in our work to be able to alter the frequency of stimulation based on the amplitude severity of the filtered LFP signal. Generally, high peaks of $\dot{x}(t)$ denote higher synchronization and HFS has been proven to have better efficiency in desynchronization [54]. However, continuous HFS increases the risk of tissue damage while decreasing the battery lifetime [55, 56]. The FAS in our proposed method tends to send HFS during the peak of $\dot{x}(t)$ and slightly decreases the frequency of stimulation as the peak of $\dot{x}(t)$ descends. This allows for enhancing the synchronization process while addressing tissue safety concerns. The amount of energy consumed by the DBS device is reduced since HFS is only used for short periods of $\dot{x}(t)$ peaks. Lower energy consumption reduces the need for costly battery replacement surgeries [57]. In addition, variant stimulation frequencies have been shown to have different therapeutic effects based on the symptoms of the patients [25, 28]. The schematic of the delayed feedback loop with the proposed FAS protocol is shown in Fig 2. In order to compare the effectiveness of the FAS method, we investigated some well-studied protocols such as Pulsatile delayed feedback [16, 17], High Frequency Stimulation (HFS) [22, 58, 59] and Variant Frequency stimulations (VFS) [29]. Similar to FAS, the Pulsatile method uses the non-linearly delayed LFP signal as the control variable, however, this control signal is used to modulate the amplitude of the DBS signal rather than its frequency [16, 17]. HFS and VFS protocols, on the other hand, work in an open loop manner where the stimulation signal is pre-defined. The traditional HFS delivers high frequency pulses (> 130 Hz) for the duration of the stimulation therapy [22, 27, 60], whereas in the VFS protocol, fixed period blocks of high and low frequency stimulations are delivered according to predefined combinations such as HFS-LFS-LFS-LFS-HFS [29].

## Results

### Firing responses

**Fig 3:**
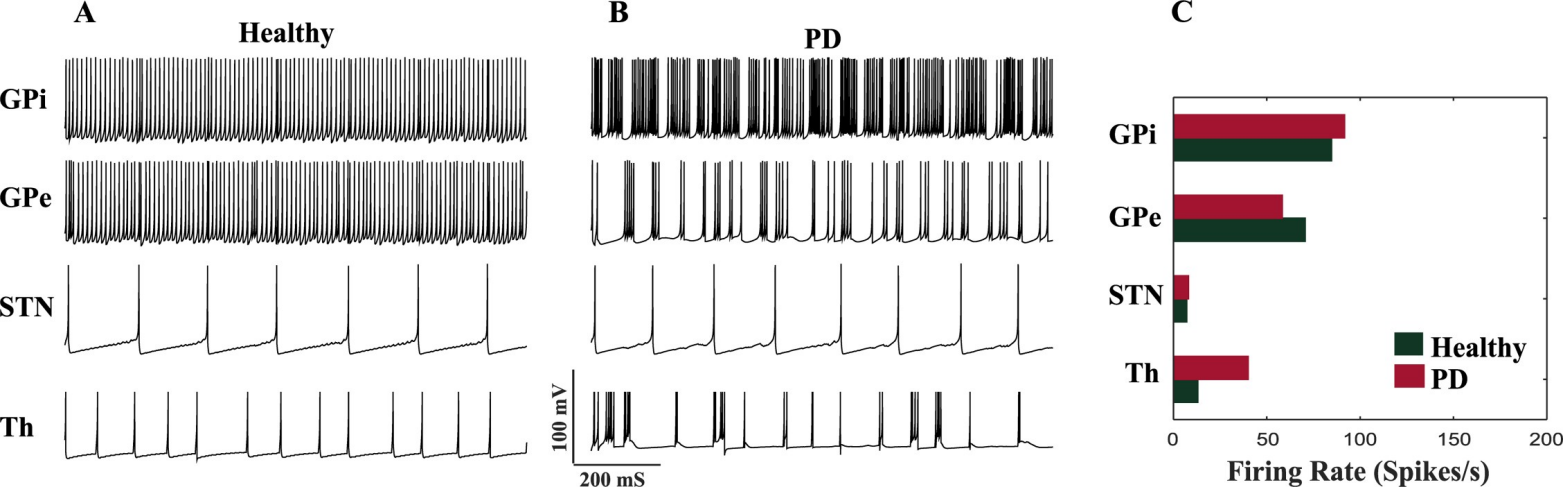
BG model validation. A) The firing patterns of 4 nuclei were generated by our proposed model under the healthy condition. Th and STN cells showed tonic spikes in presence of sensory motor cortex input, while GPe and GPi cells had continuous and repetitive firings. B) In PD state, Th cells showed abnormal firings such as burst patterns, repetitive spikes for a single stimulus and failure to fire in presence of stimulus pulses. GPe and GPi neurons showed more burst patterns while STN firings remained similar to the healthy condition. C) The average firing rate within 125 neurons of each nuclei were examined for healthy and PD states. From healthy to PD, the STN and GPi firing rates were increased, while the Th and GPe firing rates were decreased. These changes were much compatible with actual recordings [62] compared to previously proposed BG models [60, 63].

To validate the performance of the BG model in generating neuronal firing patterns, we ran the network with 125 neurons in each nucleus with all interconnections as shown in Fig 1. The firing patterns and rates were similar to the experimental recordings [40, 49] (Fig 3). In presence of sensory motor cortex input to the Th cells, unique firings are seen due to the T-type calcium currents. As shown in Fig 3, under healthy condition, the depolarizing *I*_*SMC*_ charges and discharges the Th membrane, causing a tonic pulse with each pulse of *I*_*SMC*_. Changing the model parameters to represent the PD state, contributes to the abnormal firing of the Th neurons. In Fig 3B, the Th cells show short trains of Action Potentials (APs) while missing to elicit APs at some pulses of *I*_*SMC*_. The abnormalities in Th firing patterns occur due to failure of eliciting APs when there is an input pulse, generating bursts of firings in response to a single input pulse and false spiking in the absence of any input stimulus. STN neurons had spontaneous firings at frequencies of 6 Hz and 8 Hz under healthy and PD conditions, respectively (Fig 3A and 3B). Although the healthy firing patterns match the low firing rate characteristic of the STN cells observed in [61], the STN firings frequency under PD state was lower than actual recordings (30 Hz) [62] since certain connections in our model were strengthened. Under healthy condition, both GPe and GPi neurons fire repetitive spikes, however in the PD state, the firing patterns change to tonic bursts [53, 60]. According to [62] and Fig 3C, the firing rate of the STN neurons slightly increases from healthy to PD states. Moreover, the firing rates of the GPi neurons in PD is higher than its equivalent in the healthy state. In contrast, GPe neurons fire less in PD state compared to the healthy condition. These relative alterations of firing rates from healthy to PD states in our model are more compatible with experimental recording [62], than previously proposed BG network models [60, 63].

In order to validate the dynamics of our BG model, we compared the average firing rates of STN, GPe and GPi neurons with the experimental recordings of normal (healthy) and MPTP-treated monkeys [64]. The results are shown in Table 2 and the firing rates (Spikes/s) are measured for both healthy and PD conditions. As shown in Table 2, STN neurons fired more under PD conditions which is consistent with the experimental data and previous BG models [60, 64]. Similar to the recordings of MPTP-treated monkeys, the firing rates of GPe neurons decrease under PD condition while GPi firings increase. All neuron types showed increased oscillatory behavior from healthy to PD conditions in the dominant frequency range of 8–15 Hz consistent with the experimental recordings [64]. Although the number of STN and GPe neurons oscillating at frequencies higher than 15 Hz increases from healthy to PD (or MPTP-treated), this small increase indicates a lower oscillation frequency occurring at higher beta band [65]. Additionally, the number of STN neurons showing bursting index between 8–15 Hz under PD was smaller than experimental recordings due to faster deactivation of incoming currents tuned by the model parameters, as similarly seen in biologically inspired models [60, 66]. STN, GPe and GPi cells do not show any bursting pattern at higher frequencies (> 15 Hz) in our model and the recordings [64]. For 8–15 Hz more than half of the GPi neuron population showed burst firing in PD condition similar to the MPTP recordings. Under PD state, GPe bursting patterns in 8–15 Hz were not completely consistent with recordings (Table 2), however this higher number of neurons showing burst firing was observed in many biologically inspired models [60, 66] due to the de-inactivation of the T-type calcium channels during hyperpolarization [60].

**Table 2:** Characteristics of neuronal firings.

		Proposed Method		Experimental Recordings [64]	
		*Healthy*	*PD*	*Healthy*	*MPTP*
**Spikes/S**	STN	12.5	16.7	23.2	37.3
	GPe	69.2	58.4	66.1	48.5
	GPi	76.8	85.6	73.5	78.1
**Percentage of neurons oscillating between 8–15 Hz**	STN	0.16%	63.20%	0%	50%
	GPe	12%	28%	9.10%	27.50%
	GPi	29.60%	55.20%	9.10%	50%
**Percentage of neurons oscillating higher than 15 Hz**	STN	0.80%	11.2	0%	7.10%
	GPe	3.20%	4.80%	0%	2.50%
	GPi	5.60%	4%	3%	2.90%
**Percentage of bursting neurons with 8–15 Hz oscillations**	STN	0%	9.6%	0%	21.4%
	GPe	44%	26.4%	50%	0%
	GPi	8%	53.6%	0%	52.9%

### LFP and FAS evaluation

**Fig 4:**
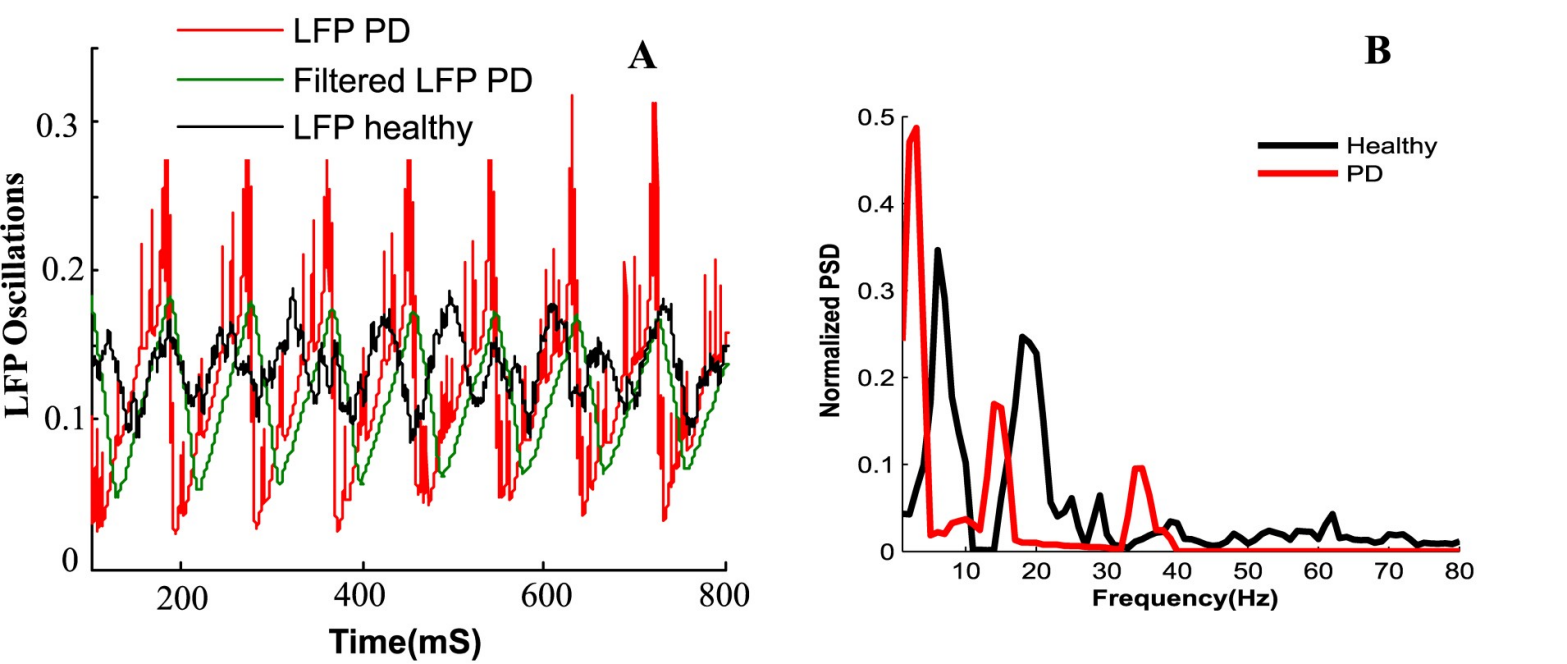
LFP measurements. The measured LFP and its filtered signal show a rhythmic oscillation due to PD.

The LFP is measured from a population of 125 STN neurons placed in a cubic area with 5 mm edges, according to Eq 10. The LFP signal is then filtered by the damped harmonic oscillator mentioned in Eq 11, to obtain $\dot{x}$. Fig 4 shows the original LFP with its filtered signal where the rhythmic behavior of the STN population is observable. The beta activity detected in the LFP correlates with the motor symptoms seen in PD [67] and measuring it can be done by the same DBS electrode or directly from the cortex [35], which makes it suitable as a feedback control variable.

**Fig 5:**
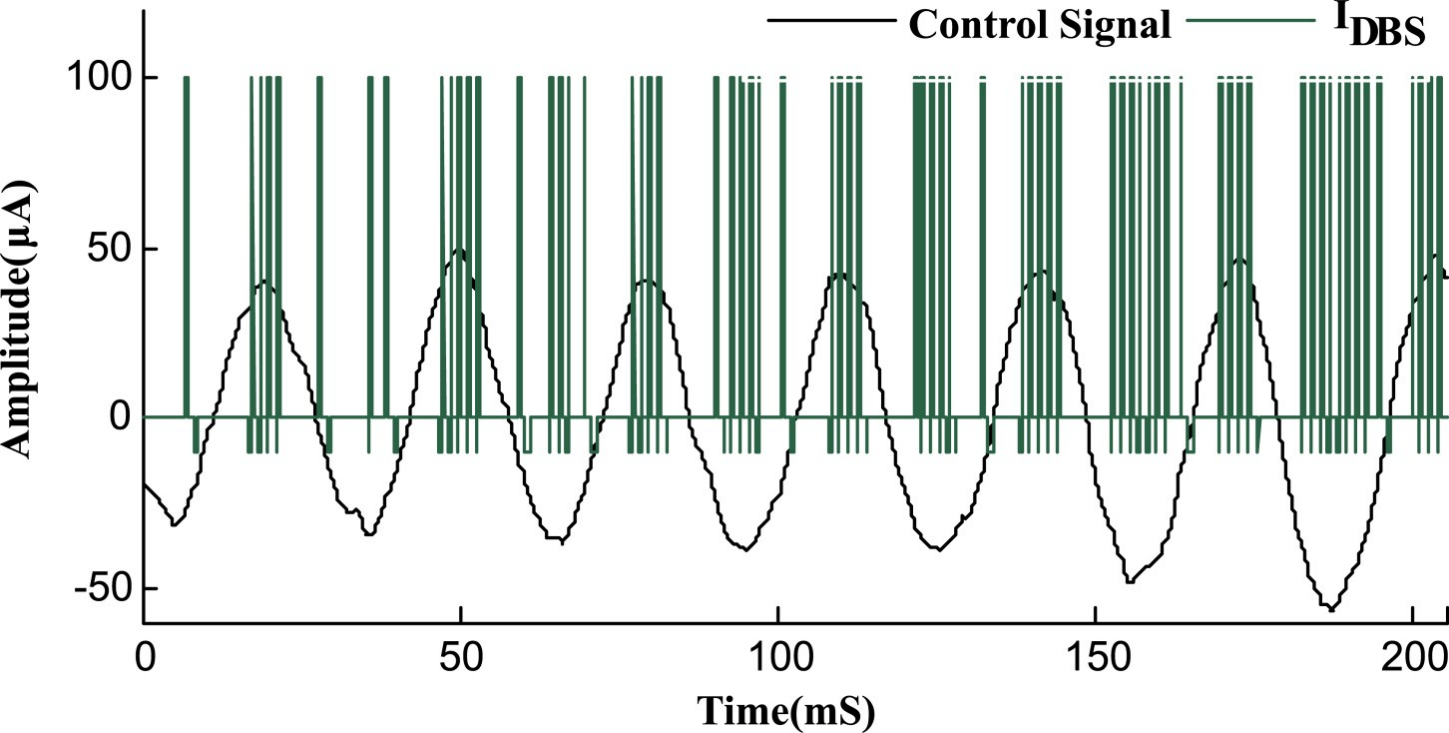
Adjusted stimulation signal by FAS protocol. The frequency of the DBS signal is modulated based on the feedback control signal (blue line). Peaks of the control signal indicate high synchronization and therefore, HFS DBS is used for maximum therapeutic effects. With lower amplitudes of the control signal, the urge for HFS decreases and *I*_*DBS*_ is then adapted to lower frequencies. The cathodic and anodic peaks of the stimulus signal were set to 100 *μA* and -10 *μA*, respectively. The control signal is magnified 100 times for better clarification.

The FAS protocol incorporates the frequency modulation of *I*_*DBS*_ according to the amplitude of the feedback signal, as illustrated in Fig 5. The adjustment of the stimulation signal *I*_*DBS*_ according to the amplitude of the feedback signal is done via Eq 12. For high amplitudes of the feedback signal, an HFS stimulation signal (130Hz) is applied and as the amplitude descends, the frequency of stimulation shifts proportionally to lower frequencies until it eventually reaches a LFS (40Hz) stimulation signal. In order to avoid an irreversible charge deposit and tissue damage [54, 55], each period of the stimulation signal concludes cathodic and anodic phases with a delay in between [8, 16, 17], as illustrated in Fig 1. This adjustment of *I*_*DBS*_ provides a charge balanced stimulus, impeding nervous tissue damages. The length of the cathodic, delay and anodic phases for the stimulus signal were set to 0.2, 0.5 and 2 *mS*, respectively, to guarantee a total charge close to zero for the biphasic stimulus pulse.

### Desynchronization of STN neurons

**Fig 6:**
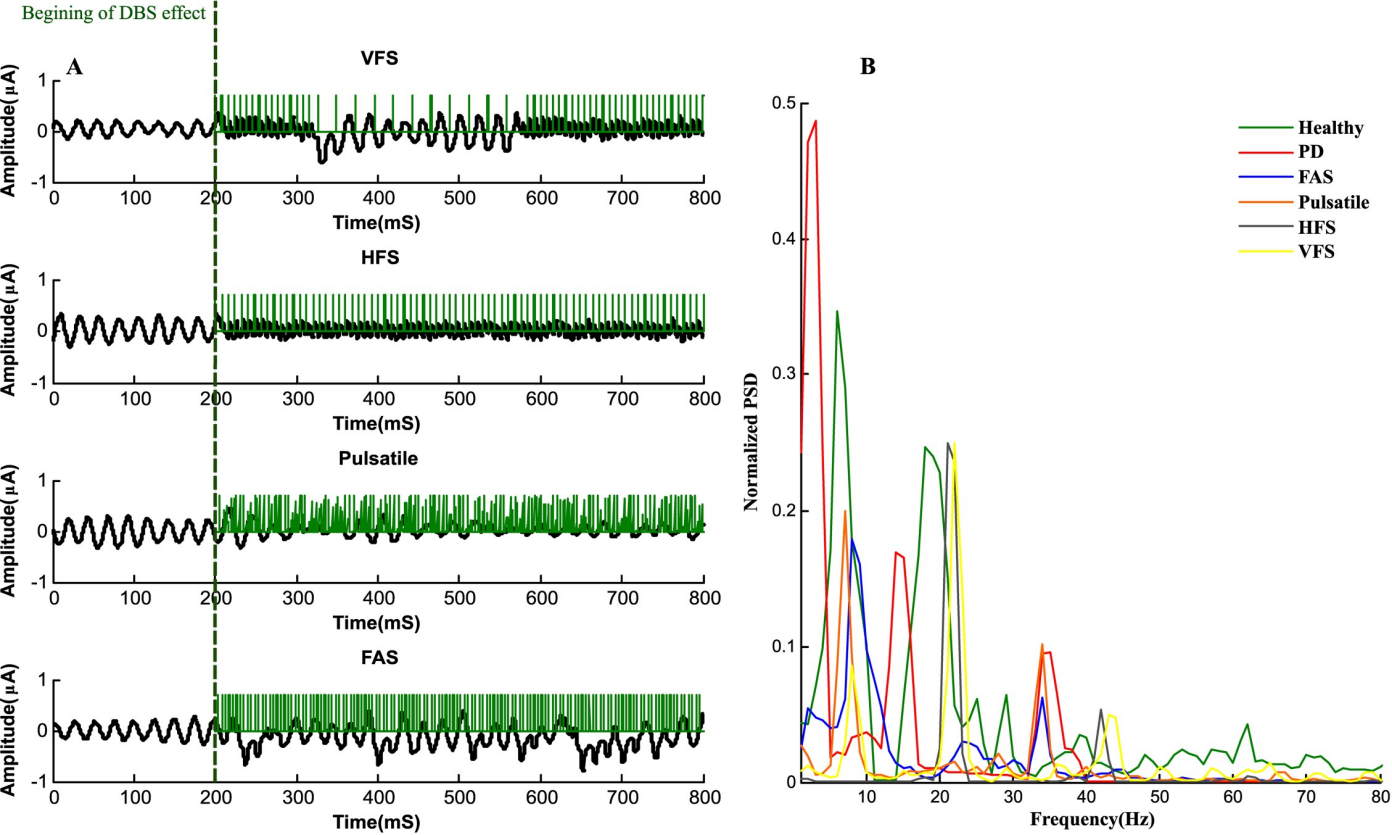
Desynchronization of STN population by various DBS protocols. **A)** DBS signals tends to abrupt the synchronization of the STN population, however, closed loop stimulation such as FAS and Pulsatile show better desynchronization effects. B) The normalized PSD of the LFP measurements for healthy, PD and different stimulation methods are shown. The LFP is down sampled and filtered using Welch’s Method and both FAS and Pulsatile were able to suppress the 14 Hz beta band oscillations, while FAS achieved better desynchronization for 34Hz oscillations. The PSD of HFS and VFS shows similar oscillation frequency with the ability to suppress the low beta band oscillations, whereas the VFS method also shows a small oscillation at 9 Hz consistent with the PSD of the healthy condition.

The FAS protocol shows a reduction in synchrony within the population of the STN neurons. We ran the network with DBS applied from the beginning, however it took 200 *mS* for the desynchronization effects to appear (Fig 6A). This delay in desynchronization is due to the STN neurons forming sub populations synchronizing in anti-phase with each other. The stimulus signal at the beginning is forced to adjust the oscillated sub population in phase with each other [33]. Finally, with in-phase oscillated neurons and sufficient amplitude of *I*_*DBS*_, the desynchronization occurs. For FAS, the sparse LFP pattern of the STN population after the initial delay of 200 *mS* in Fig 6A, shows the desynchronization capability achieved by this protocol. Another method of stimulation called Pulsatile delayed feedback [16, 17], was investigated as shown in Fig 6A. In this method, the amplitude of the stimulation signal is modulated according to the synchrony dynamics of the measured LFP. An interphase gap was also designed in the stimulus signal which provides better desynchronizing effects [16]. In contrast with the FAS and Pulsatile protocols, we studied open loop stimulation techniques such as HFS with stimulation frequency of 130 Hz and also a new stimulation method called Variant Frequency Stimulation (VFS) [29]. The VFS protocol sends the stimulus signal in fixed length blocks of different frequencies. As shown in Fig 6A, VFS applies an HFS block to the STN population, followed by two blocks of LFS and finally two blocks of HFS, again. Although VFS might be beneficial to address various symptoms associated with PD, it lacks efficient desynchronization results. The Power Spectral Density (PSD) of the filtered LFP signal under healthy and PD states are depicted in Fig 6B. Under healthy condition, the highest power occurred at 8 Hz and other peaks of the PSD were due to subsequent harmonics of the LFP signal. The PSD peaks for PD states occurred in the beta frequency range (13–35 Hz). It has been suggested that changes in the power of beta LFP oscillations might represent correlations with motor performance [68, 69]. For instance, reduction of beta band LFP power was shown to correlate with improvement in motor impairment [68, 69]. LFP oscillation in lower beta frequencies (13–20 Hz) are mainly associated with akinesia and bradykinesia, while high beta oscillations (20–35 Hz) are related to motor signs such as context recognition [70, 71]. The PSD results under healthy and PD conditions are consistent with previous studies [72, 73]. Our model was able to show a broad PSD peak at lower beta band at 14 Hz (Fig 6B). This high power spectrum reflects the synchronous dynamics of the STN neuronal population firings. Essentially, the STN neurons in PD fire with the same frequency and small delay from one another. Interestingly, the model also captured the emergent of 34 Hz oscillation (third peak of PSD under PD state in Fig 6B), which was observed previously in [65]. The STN population resonating with the GPe neurons causes the appearance of 34 Hz oscillation in PD. Both FAS and Pulsatile were able to suppress these oscillations, however the 34 Hz oscillation was more suppressed by FAS compared to the Pulsatile protocol. Also, the first peak in the PSD of the FAS and Pulsatile methods falls within the first peak of healthy PSD. FAS beta band oscillations were similar to [72, 73] where irregular or adaptive frequency stimulations are shown to suppress the high beta band oscillations better than HFS or other closed-loop stimulation methods. On the other hand, the HFS method shows to suppress the 14 Hz oscillation, however, from its PSD, it does not match the healthy conditions oscillations. The PSD of HFS shows a main peak of oscillation at 23 Hz and a smaller oscillation at 43 Hz. The 43 Hz oscillation is consistent with the results obtained in [72]. The reason for lower oscillation (23Hz) is due to parameter difference and initialization of our model. Additionally, HFS is shown to alter the intrinsic dynamics of the STN population and evoke neurons to fire at the frequency of stimulation [72]. The oscillations under VFS were similar to HFS, however, as shown in Fig 6B, there is a smaller oscillation at 9Hz consistent with the fist peak of the healthy condition. This provides an interesting hypothesis that variant frequency stimulation might adapt more to the healthy condition rather than just suppressing the beta band oscillations [29].

The other open loop technique which sends HFS pulses, provides relatively suitable desynchronization, however, HFS is shown to be less energy efficient in comparison with closed loop therapy [10]. The synchrony dynamics of the STN population was measured by the magnitude of the *LFP*_*m*_(*t*) signal and the order parameter *R*(*t*), as shown in Eqs 14–16 [16, 74].

$$R(t)=|\frac{1}{N}\sum_{j=1}^{N}e^{i\varphi_{j}(t)}|$$ (14)

$$\varphi_{j}(t_{n})=2\pi n\;for\;t=t_{n}$$ (15)

$$\varphi_{j}(t)=2\pi \frac{\left(t-t_{n}\right)}{\left(t_{n+1}-t_{n}\right)}+2\pi n\;for\;t_{n}<t<t_{n+1}$$ (16)

where *φ*_*j*_(*t*) calculates the phase of each individual neuron and *t*_*n*_ indicates the burst onsets as they appear at *n* = 0,1,2,… points in time. According to Eq 16, *φ*_*j*_(*t*) increases linearly between the consecutive bursts (*t*_*n*_,*t*_*n*+1_). The order parameter *R*(*t*) ranges from 0 (no synchrony) to 1 (absolute synchrony). Here, we defined a Synchrony Index (SI) that incorporates the phase calculation done by the order parameter with the magnitude of the *LFP*_*m*_(*t*) signal. Since high peaks of *LFP*_*m*_(*t*) represent high synchrony, we multiply *R*(*t*) by the normalized *LFP*_*m*_(*t*) amplitude and then the average of the obtained signal over time is used as the SI value, as stated in Eq 17.

$$SI=\frac{1}{L}\sum_{t=0}^{L}R(t)||{LFP}_{m}(t)||$$ (17)

The result examines the amount of synchrony between 0 and 1 which corresponds to the absence and presence of full synchrony, respectively. In Table 3, we showed the mean of the order parameter *R*(*t*) and *LFP*_*m*_(*t*) signals over a period of 1 *S* for all protocols shown in Fig 6A. We also measure the SI for a more comprehensive examination of synchrony. As can be seen in this table, the closed loop stimulation techniques (FAS and Pulsatile) demonstrated lower $\bar{R(t)}$ in comparison with open loop methods (HFS and VFS). However, the amplitude of the *LFP*_*m*_(*t*) signal was lower in cases of HFS and Pulsatile stimulations. According to the SI values in Table 3, FAS and Pulsatile provided the best desynchronization, while traditional HFS or VFS protocols were less successful in desynchronization. Furthermore, we measured the percentage of the activated STN neurons by each stimulation protocol. As shown in Table 3, the FAS method was able to activate 95.2% of the STN neurons which was the highest amount in comparison with other techniques. This shows that amplitude modulation used in other protocols such as Pulsatile delayed feedback [16, 17] might decrease the efficiency of stimulation in terms of the total number of activated cells. In contrast, the frequency modulation done by the FAS protocol provides the highest neuronal activation.

**Table 3:** Synchrony measures for different stimulation protocols.

Protocol	$\bar{R(t)}$	$\bar{{LFP}_{m}(t)}$	SI	Neuronal Activation
FAS	0.53	0.88	0.47	95.2%
Pulsatile	0.61	0.84	0.52	84.4%
HFS	0.66	0.85	0.56	85.6%
VFS	0.69	0.89	0.61	88.8%

The highest desynchronization based on the SI was achieved by the FAS protocol, while obtaining the maximum number of STN neurons activated.

**Fig 7:**
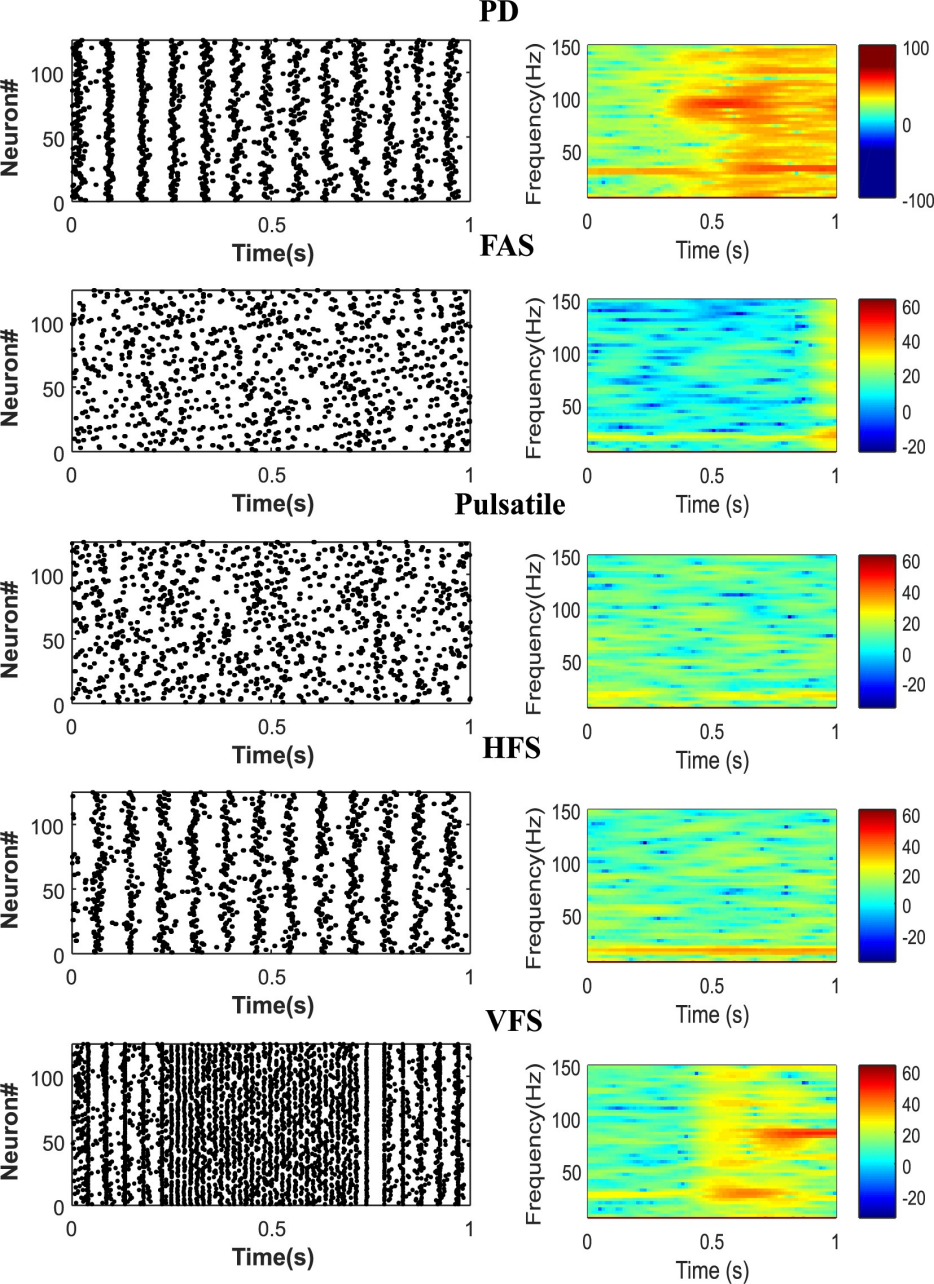
Closed and open loop protocols in desynchronizing STN neurons. A) Synchronous behavior observed under PD condition in raster plot (left panel) and spectrogram (right panel). B) The spectrogram of synchronization while FAS was applied was the lowest, indicating the capability of frequency modulated protocols. The mixture of responses in neuronal firings was prevailing in the FAS protocol (left panel). C) The Pulsatile method also achieved great desynchronization results and the neuronal firings were observed to be sparse. Open loop stimulation methods such as HFS and VFS (D and E, respectively) showed semi-synched dynamics in the firing patterns (left panels). Also, the VFS method showed high synchronization at 100 Hz, as it lacks a precise method defining the length of each stimulation block.

The dynamics of PD in our model are shown through the spectrogram and raster plots of 125 STN neurons (Fig 7. A right). We can observe a high synchronization at low frequencies in the spectrogram of PD which is a significant property of pathological networks [53]. Applying the DBS currents shows desynchronizing effects particularly at the low frequencies, as shown via the spectrograms of Fig 7B–7E. Comparing the spectrograms of 4 different stimulation protocols, we conclude that all stimulations were able to desynchronize the network at low frequencies, however closed loop FAS and Pulsatile methods were more effective (Fig 7B and 7C). It was also observed that the power densities depicted by the spectrograms were more spread, which is consistent with patterns seen in patients undergoing L-Dopa treatments [75]. According to the power density scale shown in the color bars of Fig 7, the FAS protocol achieves the highest desynchronization of the STN population. As shown in the raster plots of Fig 7, neuronal firings under DBS tends to show a mixture of responses over time. The increased, decreased or stabled firing rates is believed to be a part of the DBS therapy [76]. Due to the orthodromic modulation by the DBS signal, these mixture of firing rates happen in the STN population [73]. This mixture of responses from the STN to GPi neurons might balance the regularization and inhibition of the GPi cells [76]. The FAS and Pulsatile protocols show better mixture of responses in comparison with HFS and VFS (Fig 7D and 7E left). However, the adaptive frequency stimulation in FAS or VFS methods might be more beneficial in terms of addressing various PD symptoms [29]. In addition, the VFS method showed high synchronization at 100 Hz (Fig 7 E), which is due to the fact that it lacks a precise method for defining the length of each stimulation block.

### Energy consumption

**Fig 8:**
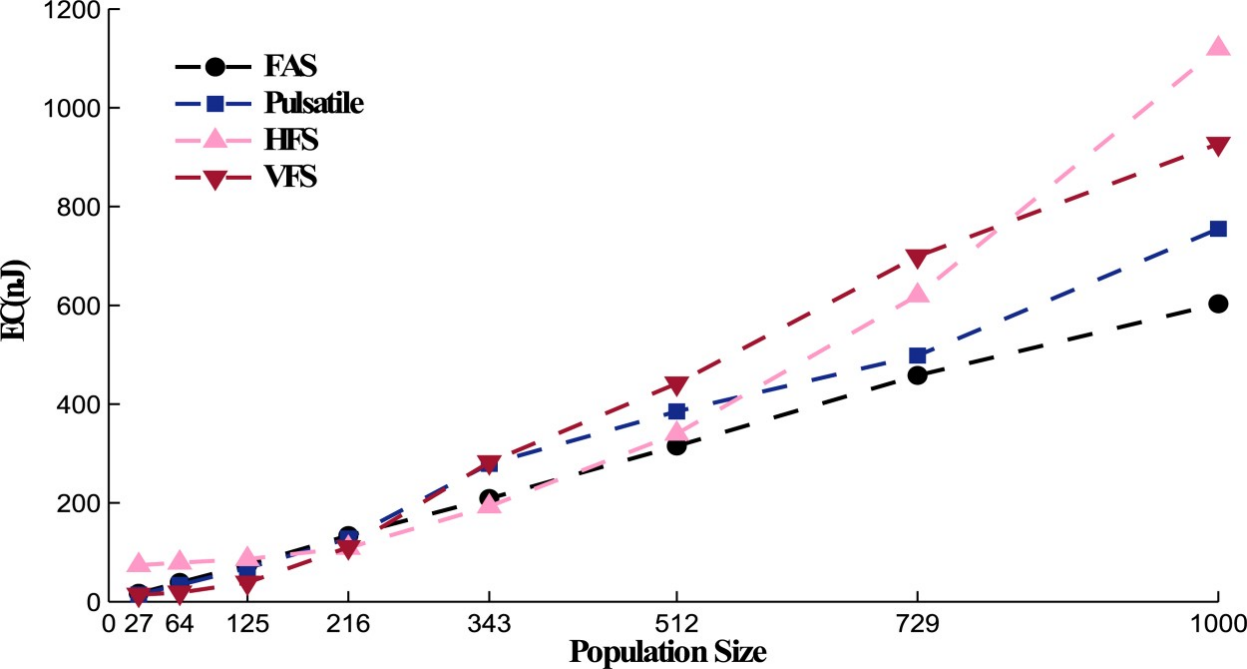
Population size effect on the total energy consumption. As the network size increases, the EC value for FAS and Pulsatile protocols ascend linearly with a moderate slope. For big networks (>512 neurons in each nucleus), FAS shows to be more energy efficient than the Pulsatile stimulation. The EC for open loop stimulation therapies such as HFS protocol grows almost exponentially as the population gets bigger. VFS was able to show a linear growth in EC as the population size increases, however, it drastically became less energy efficient for medium and big populations (>216 neurons in each nucleus).

In the FAS protocol, the ability to send HFS whenever needed provides lower energy consumption in comparison with previous stimulation methods [16, 17, 29, 53]. According to [8–10], the total energy consumed by the DBS signal is measured as follows.

$$EC=\int_{0}^{PW}{I_{DBS}(t)}^{2}Z(t)dt+M$$ (18)

where *Z*(*t*) is the constant impedance set to 1*k*Ω, *PW* is the width of the DBS waveform. And *M* denotes the number of misses in neuronal activation or eliciting action potentials. Every time a DBS pulse is applied to the STN population, we measure the number of neurons that elicit action potentials. If this number is less than 70% of the whole population, we set *M* to 1 and consider the DBS pulse as a miss or unsuccessful stimulation (each miss is considered with the penalty of 2 nJ). The amount of energy consumed by *I*_*DBS*_ in the FAS protocol was 42% less than its equivalent traditional HFS method. Comparing energy efficiency of the FAS protocol with the Pulsatile method [16, 17] reveals a slight difference. The total amount of energy consumed for 1 *S* stimulation of a population of 125 STN neurons was 75 nJ and 68 nJ under the FAS and Pulsatile protocols, respectively. However, FAS was able to stimulate more neurons than the Pulsatile method, as stated in Table 3. Since the FAS method maintain a constant amplitude for stimulation, the chance of neuronal activation is higher in comparison to Pulsatile stimulation where the amplitude modulation causes less neuronal activation. Also, for highly synchronized networks reducing the stimulation amplitude shows a reversing effect and increases the oscillations [34]. Other frequency methods such as VFS [29] also show low energy consumption (80 nJ), however they provide lower neuronal activation and desynchronization. The reason why VFS protocol is energy efficient is due to the LFS blocks that target the STN population. Generally, sending pulses with lower frequencies (LFS) guarantees less energy consumption. The blocking protocol of high and low frequency stimulation might be beneficial in the treatment of certain symptoms of PD such as postural instability, gait dysfunction and speech problems [29, 77, 78]. However, their low desynchronization effect has inspired this research for devising the FAS protocol with more control over the frequency of stimulation. In Fig 8, we studied the effect of the network size on the total energy consumed. The Energy Consumed (EC) was obtained according to the integration of the instantaneous power of the DBS signal over time, according to Eq 18 [8, 9]. As shown in Fig 8, the EC value under the FAS protocol increased linearly with the population size. Since the neurons in our model are arranged in a cubic area, the network size takes a cubic form. The smallest population was set at 27 neurons (a cube of 3 neurons in each edge, as shown in Fig 1) and the biggest population size was consisted of 1000 neurons in a cubic placement. From Fig 8, we can conclude that the population size has lower effect on the EC value for FAS and Pulsatile protocols in contrast with HFS, where EC grows exponentially as the population increases. VFS consumes high energy for relatively large population sizes (>343 neurons in each of the four different nuclei), which make it less practical for patients with severe symptoms. FAS, being the most energy efficient protocol, can maintain longer battery lifespan and therefore reduces the costly battery replacement surgeries [13, 55, 79].

## Discussion

In this study, we developed a computational model of four nuclei within the basal ganglia according to the reduced order model of Izhikevich [39]. The synaptic connectivity within each nucleus and with other cell types were adjusted using both physiological and mathematical representations. This significantly reduced the computational cost, while reliably capturing the neural activations and LFPs. The lower computational cost provided the opportunity to investigate the effect of DBS on large-scale networks. However, the computational models such as the one developed in this paper do not represent the whole complexity of physiological systems [80–82]. For instance, our model does not consider the direct projections from the Th cells to STN cells [83]. Although we defined a sensory motor cortex current into the network, the cortical role in the synchrony of STN neurons [84] was not fully represented by this model and the non-somatic effect of DBS is not fully examined by computational models to date. Moreover, the changes of the STN neuronal activity seen in PD does not completely reflect the Thalamo-Cortical level, which is difficult to be produced by models [25]. The mechanism of beta-band oscillations appearance is more complicated in physiology than computational models. Beta-band oscillations are not easily detectable by all patients, suggesting implementation of two sub-bands which carry more information and can be more dependable biomarkers of PD [35]. Another drawback of our model is that it cannot record the LFP focally [85]. However, since we only examined the effect of the FAS protocol on the LFP of the STN population, focal measurements of LFP is not essential. Finally, it is still not clear if the LFP alone can be a suitable control variable for the closed loop stimulation since it might not be observed in all patients [35, 86]. On the other hand, it’s been shown recently that interactions of various oscillations observed from various targets within the basal ganglia might reveal more information rather than the measured LFP [87].

Our model was able to generate the beta-band oscillations at 34 Hz with the burst firings of the STN neurons under PD or dopamine depletion. As shown in [34], strong oscillations in PD appear as soon as the amplitude of the stimulation decreases. Therefore, here, we maintained a certain amplitude (100 *μA*) while adjusting the frequency to send HFS only when there is strong coupling.

The FAS protocol in this research was incorporated in a delayed feedback closed loop manner. Both open loop and closed loop high frequency stimulations might show similar results since the signal generation circuitry is very similar [34]. However, adapted signals in a delayed feedback method can reduce the side effect of tissue damage, enhance the desynchronization performance and increase the battery life [11, 32, 88]. Also, closed loop stimulation is superior to open loop in terms of alleviating the motor symptoms and desynchronization [33]. Furthermore, as the beta-band oscillation does not appear consistently, closed loop stimulation of the beta-band with more pulses at higher synchronization and less at lower synchronization is more efficient than the traditional open loop stimulations [89]. The LFP oscillations in PD are associated with the neural activity of STN [90], which makes them applicable as the controlled variable.

The LFP recording in our model was filtered by a damped harmonic oscillator and then linearly delayed, which has shown to be more effective in desynchronization. The outcome is used as the control signal which provides the charge balanced properties of HFS along with the desynchronization efficiency of the delayed feedback signals [91]. The FAS protocol tends to send HFS during the peaks of the control signal which enhances desynchronization, while being reliable on tissue safety concerns. Also, as previously shown in [8, 16, 17], the interphase delay (Fig 1) in the stimulation signal significantly improves the desynchronization process in a delayed feedback protocol. In addition, longer delays reduce the need for higher amplitude of stimulation, contributing to prolonged battery life [8].

In summary, the FAS protocol has shown to be more efficient in the suppression of the STN oscillations along with generating a mixture of firing responses, which has been associated with the efficacy of DBS [73, 76]. Moreover, we suggest that the FAS protocol could better control multiple symptoms of PD if the appropriate targets for stimulation are selected rather than Pedunculopontine Nucleus (PPN) which is used for patients with gait dominant problems. Additionally, the delayed feedback FAS protocol was more energy efficient compared to other stimulation methods. For instance, the EC for the FAS protocol in a relatively large network of 1000 neurons, in each nucleus was 16.1%, 74% and 44% less than Pulastile, HFS, and VFS protocols, respectively (643 nJ for FAS compared to 750 nJ, 1120 nJ, and 926 nJ). Finally, the feedback stimulation by FAS was able to activate larger regions of the STN populations, which is crucial in large-scale network simulations [92]. All of these benefits that a frequency modulation in FAS protocol provides, opens the path towards more algorithms to tackle DBS therapy in the future by various modulations in stimulation that is administered on demand or based on a delayed feedback.

## Supporting information

S1 File Neuronal dynamics, firing patterns, LFP recordings, PSD and EC data. (PDF)
