# Supplementary material for: Robust desynchronization of Parkinson’s disease pathological oscillations by frequency modulation of delayed feedback deep brain stimulation
Source: PLoS One. 2018 Nov 20;13(11):e0207761. doi: 10.1371/journal.pone.0207761 (PMC6245797; doi:10.1371/journal.pone.0207761)
Supplement: S1 File — (PDF) [file pone.0207761.s001.pdf]

Supporting Information. Neuronal Dynamics, firing patterns, LFP recordings, PSD and EC data.

Neuronal firings in this work were generated through modified differential equations stated in the manuscript. Table 1 in the manuscript shows the parametrization for these equations. The results of generated data can be found on:

https://figshare.com/articles/supporting_data_mat/6177269.

All the generated data in this work are labeled according to Table S1 and are accessible via the above link.

Table S1. Generated Data

	Neuron type	Corresponding equations	Matrix Label	Matrix size
**1**	Th Firings/Healthy	(5)	VVth1	27×100001
**2**	STN Firings/Healthy	(7)	VVstn1	27×100001
**3**	GPe Firings/Healthy	(8)	VVgpe1	27×100001
**4**	GPi Firings/Healthy	(9)	VVgpi1	27×100001
**5**	Th Firings/PD	(5)	VVth2	27×100001
**6**	STN Firings/PD	(7)	VVstn2	27×100001
**7**	GPe Firings/PD	(8)	VVgpe2	27×100001
**8**	GPi Firings/PD	(9)	VVgpi2	27×100001
**9**	LFP recordings	(10)	LFP_stn	1×100001
**10**	Filtered and delayed LFP	(11) and (13)	LFP_m	1×16401
**11**	Frequency modulated DBS signal	(12)	I_DBS	1×16401
**12**	PSD of STN population/Healthy		PSD_H	8193×1
**13**	PSD of STN population/PD		PSD_PD	8193×1
**14**	PSD of STN population/FAS		PSD_FAS	8193×1
**15**	PSD of STN population/Pulsatile		PSD_Pulsatile	8193×1
**16**	PSD of STN population/HFS		PSD_HFS	8193×1
**17**	PSD of STN population/VFS		PSD_VFS	8193×1
**18**	Energy Consumed/FAS	(18)	EC_FAS	1×1001
**19**	Energy Consumed/Pulsatile	(18)	EC_Pulsatile	1×1001
**20**	Energy Consumed/HFS	(18)	EC_HFS	1×1001
**21**	Energy Consumed/VFS	(18)	EC_VFS	1×1001

The labels of firing patterns under healthy and PD conditions of all cell types are shown in rows 1 through 8. Each nucleus is represented by a matrix of 27*100001, where each row represents one neuron and each column is the membrane voltage of that neuron at a specific time. The recorded LFP signal from the population of STN neurons is shown in row 9 and then this signal is filtered and delayed to define the control variable in row 10. The frequency of the DBS signal

is modulated according to the amplitude of the control signal (LFP_m) and the label of I_DBS can be seen in row 11. For clarity, only 16401 samples of LFP_m and I_DBS are shown. The power spectral densities (PSDs) of the LFP signal under various conditions are shown in rows 12 until 17. Finally, the energy consumed under different stimulation protocols are shown in rows 18 to 21.
